# Feasibility study of cryoballoon ablation for atrial fibrillation with KODEX-EPD: a single center experience

**DOI:** 10.1038/s41598-023-49475-6

**Published:** 2023-12-22

**Authors:** Subinuer Wubulikasimu, Liang Wang, Suxia Yang, Wanyue Sang, Yafan Han, Lu Wang, Feifei Wang, Xianhui Zhou, Jianghua Zhang, Qiang Xing, Zukela Tuerhong, Jiasuoer Xiaokereti, Yankai Guo, Baopeng Tang, Yaodong Li

**Affiliations:** 1 The First Affiliated Hospital of Xinjiang Medical University, Urumqi, China; 2 Department of Pacing and Electrophysiology, Department of Cardiac Electrophysiology and Remodeling, Urumqi, China

**Keywords:** Randomized controlled trials, Interventional cardiology, Cardiac device therapy

## Abstract

To evaluate the feasibility of cryoballoon (CB) ablation of atrial fibrillation (AF) under the guidance of a new three-dimensional (3D) mapping system KODEX-EPD. 40 patients scheduled for CB ablation of AF in the first affiliated Hospital of Xinjiang Medical University from August 2021 to July 2022 were randomly divided into two groups: KODEX-EPD 3D mapping system guidance group (KODEX group, n = 20) and conventional two-dimensional perspective group (standard group, n = 20). The ablation time, operation time, fluoroscopy time, fluoroscopy dose, contrast agent dosage and follow-up data were compared between the two groups. Besides, the feasibility and accuracy of the dielectric sensing system in evaluating pulmonary vein (PV) occlusion in patients with AF during CB ablation were verified. All pulmonary veins were being isolated. The ablation time (36.40 ± 6.72 min vs 35.15 ± 6.29 min, P > 0.05) and the operation time (64.20 ± 11.82 min vs 66.00 ± 13.18 min, P > 0.05) were not statistically different in the two groups. The standard group has longer fluoroscopy time, dose and contrast medium dosage. There were significant differences in fluoroscopy time (532.30 ± 72.83 s vs 676.25 ± 269.33 s, P < 0.05), fluoroscopy dose (110.00 ± 28.64 mGy vs 144.68 ± 66.66 mGy, P < 0.05), and contrast medium dosage (71.90 ± 5.97 ml vs 76.05 ± 5.93 ml, P < 0.05) between the two groups. The learning curves of the first 5 patients and the last 15 patients in the KODEX group were compared. There was no statistical difference in the ablation time (36.80 ± 8.56 min vs 36.27 ± 6.34 min, P > 0.05) or the operation time (69.00 ± 5.00 min vs 62.60 ± 13.10 min, P > 0.05); however, compared to the first 5 patients, fluoroscopy time (587.40 ± 38.34 s vs 513.93 ± 73.02 s, P < 0.05), fluoroscopy dose (147.85 ± 35.19 mGy vs 97.39 ± 8.80 mGy, P < 0.05) and contrast medium dosage (79.60 ± 1.14 ml vs 69.33 ± 4.45 ml, P < 0.05) were significantly decreased. Using pulmonary venography as the gold standard, the sensitivity, specificity of the completely occlusion in KODEX group was 93.6% (95% CI 85–97.6%) and 69.6% (95% CI 54–81.8%); and the sensitivity, specificity of the small leak in KODEX group was 93.1% (95% CI 82.4–97.8%) and 82.0% (95% CI 65.9–91.9%). During an average follow-up of (9.90 ± 1.06) months, there was no statistical difference in arrhythmia recurrence and antiarrhythmic drugs taking after CB ablation between the two groups (P > 0.05). Using the KODEX-EPD system, the CB ablation procedure can correctly evaluate the PV occlusion, and significantly reduce fluoroscopy exposure and contrast medium without significantly increasing the operation time.

## Introduction

Atrial fibrillation (AF) is one of the most common arrhythmias in the clinic, which is related to the increased risk of death, stroke, and peripheral vascular embolism. It is estimated that by 2050, China will have 5.2 million men over 60 years old and 3.1 million women with AF, which is 2.3 times the projected prevalence rate in the United States^1,2^. Therefore, timely and effective treatment of AF is critical. At present, the treatment of AF is constantly evolving, and CB ablation has drawn increasing amounts of interest as a relatively new catheter ablation technique^3,4^.

With the continuous expansion of clinical indications and continuous updating of clinical evidence, catheter ablation of AF has been regarded as a first-line rhythm control therapy to improve symptoms in patients with symptomatic AF. According to the current 2020 ESC/EACTS guidelines^5^, catheter ablation of AF with paroxysmal AF is recommended with class B of IIa evidence, while persistent AF without major risk factors for recurrence of AF is recommended with class C of IIb evidence. Pulmonary vein isolation (PVI) is the cornerstone of AF catheter ablation. Complete isolation of pulmonary veins (PVs) should be achieved in all AF catheter ablation operations. CB ablation has become one of the most widely used methods for achieving PVI. Phrenic nerve injury (PNI) is one of the most common complications in the process of CB ablation. Persistent AF, large PV ostia, and deep balloon position under fluoroscopy are associated with a high incidence of PNI^6^. The key to reducing the risk of PNI is to keep the balloon position as flat as possible and to ablate the vestibular side of the PV as far as possible. It is recommended to use the proximal sealing technique (PST) to prevent any deep CB position, isolate the larger vestibular ring of PV, and decrease the incidence of PNI^7^.

The proximal sealing technique (PST) can ensure that the cryoballoon (CB) is ablated in the vestibule of PV, which is safer and more effective^8^. This method requires the use of a contrast agent under the guidance of conventional fluoroscopy to confirm that the balloon is completely blocked, and then withdraws the balloon, causing the contrast agent to leak appropriately and allowing the location of the PV ostial, to achieve proximal ablation. PST is only a standard operation flow in the current literature, and there is no relevant judgment standard, which creates some barriers to the remarkable promotion of this method.

**Figure 1:**
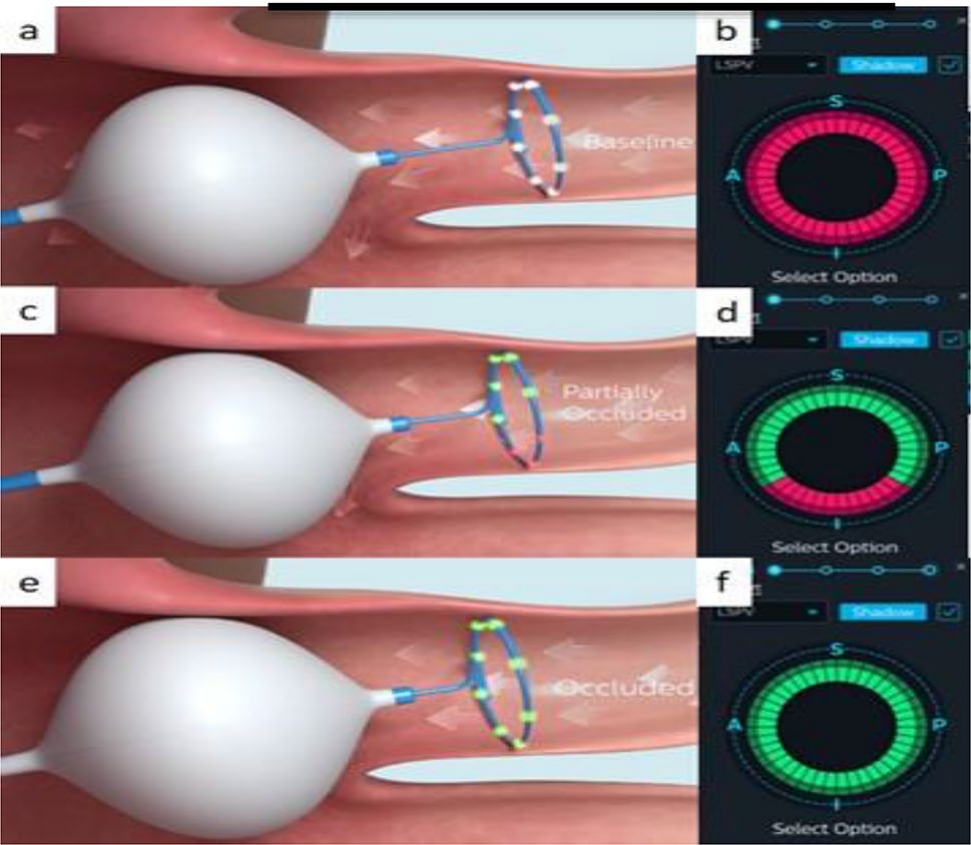
Baseline tool of KODEX-EPD system: (**a**,**b**) achieve enters the PV and the balloon is not attached to the PV ostium. (**c**,**d**) The balloon is advanced against the PV ostium with partially occlusion of PV; (**e**,**f**) complete occlusion of PV.

**Figure 2:**
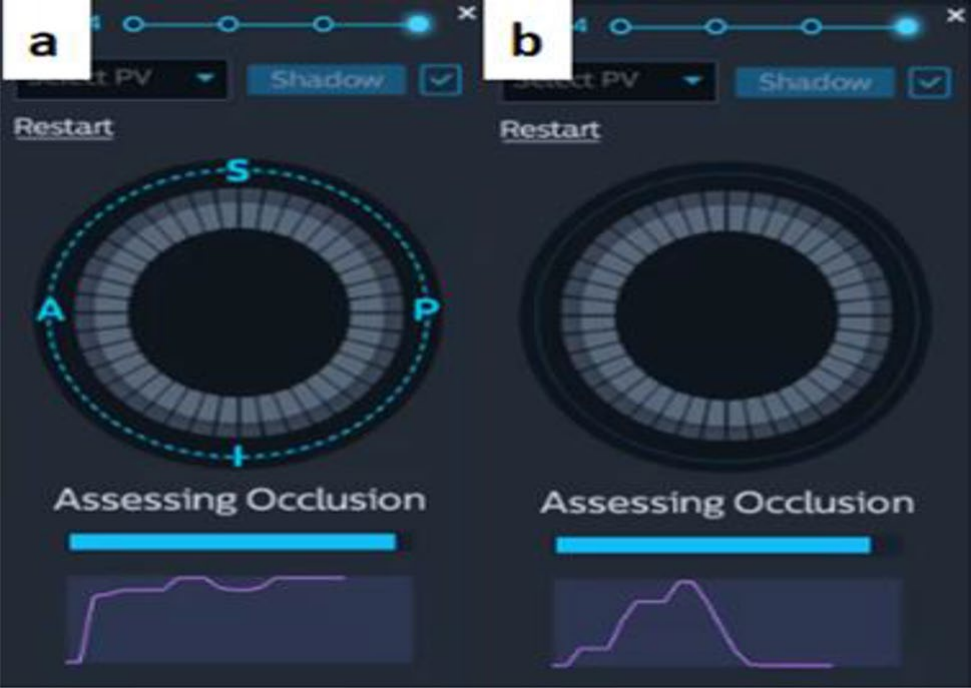
Injection tool of KODEX-EPD system: (**a**) optimal occlusion; (**b**) large leakage of contrast medium.

**Figure 3:**
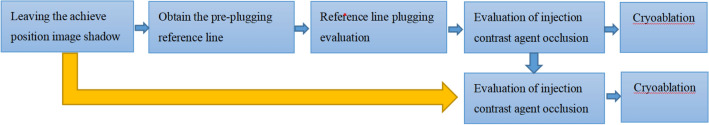
The flow chart of CB ablation surgery under the guidance of KODEX-EPD.

The KODEX-EPD system (EPD Solutions, a Philips Company), as a novel, dielectric three-dimensional mapping system, has recently gained widespread attention because of its unique occlusion verification software to facilitate CBA procedure^9–11^. The system can create high-resolution images of a cardiac structure during CB ablation, accurately visualize the anatomical structure of PV, and evaluate PV occlusion using baseline tool and injection tool. The baselines tool can determine the occlusion of the balloon in real-time by comparing the changes in the electric field of each electrode on the Achieve electrode before and after the balloon push (Fig. 1). The injection tool is that after balloon occlusion, compared with the contrast medium injection, the overall qualitative state of the voltage around the Achieve catheter is displayed to judge the balloon occlusion (Fig. 2). The two tools can be used sequentially or separately (Fig. 3), with the baseline tool judging the balloon-plugging situation and the injection tool verifying it. Previous studies^12–14^ have pointed out that CB ablation of AF under the guidance of the KODEX-EPD 3D system is desirable, which can provide real-time feedback on the degree of PV occlusion during CB ablation and reduce intraoperative radiation exposure.

In this paper, a randomized controlled study was conducted in 40 consecutive patients with the indication of CB ablation of AF in a single center to investigate and evaluate the feasibility of CB ablation guided by the KODEX-EPD system. Furthermore, we intend to assess the feasibility of KODEX guided PST in CB procedures.

## Methods

### Study population

Patients scheduled for CB ablation of AF in the Department of Cardiac pacing in the first affiliated Hospital of Xinjiang Medical University from August 2021 to July 2022 were randomly divided into the standard group and the KODEX group. All patients understand the indications and risks of the operation before the operation, and sign the operation written consent form. And informed written consent has been obtained from all participants and/or their legal guardians. The same operator team performed CB ablation of AF on all 40 patients. The baseline characteristics of patients, procedure data were analyzed. Patients with previous ablation history, significant valvular abnormalities, and intracardiac thrombus were excluded from the study. Inclusion criteria: patients with paroxysmal AF or persistent AF whose duration is less than 12 months, according to the existing quality regimen, patients with AF need CB ablation: the main inclusion conditions are: (1) 18 years old ≤ age ≤ 85 years old; (2) symptomatic patients with drug-refractory paroxysmal or persistent atrial fibrillation; (3) patients who understand the purpose of the research, voluntarily participate in the research and sign the informed written consent are suitable. Exclusion criteria: those with any of the following criteria were excluded: (1) valvular AF or AF secondary to electrolyte disorders, thyroid disease, or other reversible non-cardiac causes; (2) previous history of left atrial ablation or surgery (including left atrial appendage occlusion); (3) left ventricular ejection fraction (LVEF) < 40%, or NYHA cardiac function grade III and IV or left atrial diameter ≥ 55 mm; (4) left atrial or left atrial appendage thrombus; (5) moderate to severe left ventricular hypertrophy (wall thickness > 1.5 cm); (6) patients with hemorrhagic stroke within 3 months; (7) patients with severe liver and kidney disease (ALT ≥ 3ULN or eGFR < 50 ml/min); (8) patients with obvious bleeding tendency and hematological diseases; (9) patients with acute coronary syndrome or patients who need stent implantation; (10) the participants have participated in clinical trials of other drugs or devices in the same period.

### CB ablation procedure

All patients received a detailed preoperative evaluation, including medical history, physical examination, necessary laboratory examination, 12-lead ECG, cardiac ultrasound, left atrial and PV 3D reconstruction CT examination to understand the anatomical structure of the left atrium and PV. Left atrial appendage thrombus was excluded by transesophageal 3D echocardiography. All patients knew the indications and risks of the operation before the operation and signed the operation written consent form. The flow chart of this study is shown in Fig. 5.

All the procedures were conducted in local anesthesia with mild sedation. After access to the LA with the Brockenborough needle and an 8.5-French Swartz sheath (SL1; St. Jude Medical, MN), a 28-mm CB catheter (Arctic Front Advance; Medtronic Inc., MN) was introduced into the LA via a 12F steerable sheath (FlexCath; Medtronic Inc.).

#### Standard group

The achieve electrode was sent into the target PV for recording the PV potential, the CB was inflated in the left atrium, the filled CB was pushed along the Achieve electrode to the target PV orifice for occlusion, and the contrast medium was injected into the hollow lumen of the CB catheter, and then observed under fluoroscopy. If there is no contrast medium leakage into the left atrium, the balloon is considered to be well-occluded. PST is used to retreat the CB properly, observe the slight leakage of the contrast medium, and determine that the CB is located in the vestibule of the PV. When the second-generation balloon is used for freezing, the freezing time is 180 s, one freezing/thawing cycle at a time. If the balloon temperature is lower than − 55 °C, the freezing will be terminated immediately. After each freezing, achieve electrode was used to evaluate the electrical isolation of PV. CB ablation is usually performed in the order of left superior pulmonary vein (LSPV), left inferior pulmonary vein (LIPV), right superior pulmonary vein (RSPV), and right inferior pulmonary vein (RIPV). To prevent phrenic nerve paralysis (PNP), during CB ablation of the right pulmonary vein, the 4-pole electrode was sent to the superior vena cava to continuously pace the phrenic nerve. The assistant placed the hand on the patient's abdomen or observed the diaphragm movement under fluoroscopy. If it is found that the diaphragm movement weakens or disappears, it is necessary to stop freezing immediately. The endpoint of the operation was the electrical isolation of all PVs. If the electrical isolation of PV cannot be achieved after freezing 3–4 times, a radiofrequency (RF) ablation catheter should be used for supplementary ablation.

#### KODEX group

Mapping of the LA and PVs was performed with a circular mapping catheter (Achieve; Medtronic Inc.). The LA was reconstructed using the mapping catheter with the guidance of the 3-dimentional navigation system (KODEX-EPD Navigation system, version 1.4.8a). The anatomic sites of LSPV, LIPV, RSPV, RIPV, and superior vena cava (SVC) were marked. Then the Achieve electrode was sent into the target PV for mapping and recording PV potential. The CB was inflated in the left atrium, and the CB was pushed along the Achieve electrode to the target PV orifice for occlusion.

**Figure 4:**
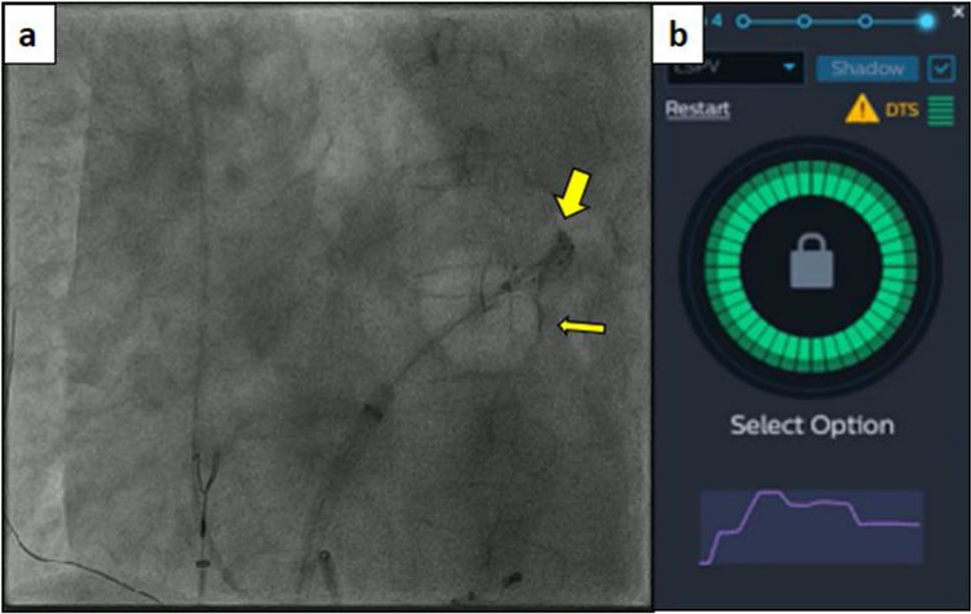
PST under the guidance of KODEX-EPD: (**a**) retention and leakage of contrast medium in PV under X-ray; (**b**) changes of dissipation curve of KODEX-EPD injection tool.

The KODEX Occlusion assessment tools include the baseline tool and injection tool. In the baseline tool, the occlusion status was real-timely visualized as a colored-wheel in which green indicated optimal contact whereas red indicated sub-optimal contact. In injection tool, the occlusion status was depicted through the injection curve after a single injection of contrast. Optimal occlusion was achieved if the last quarter of the injection curve remained in the upper half. Otherwise, various leaks might be present. When multiple red electrodes show partial occlusion, balloon operation can be displayed in real-time to achieve better and complete occlusion without multiple injections of contrast agent and angiography. After all the green electrodes showed by the baseline tool, the proximal occlusion was evaluated by injection and X-ray, and CB ablation was performed after meeting the conditions. Among them, under the guidance of KODEX-EPD, the performance of PST is mainly presented by injection tool. The injection tool's dissipation curve is divided into three segments (Fig. 4), the first of which climbs rapidly, the second of which can maintain a high position or decreases with a lower slope, and the third of which decreases slowly and eventually stabilizes at about half of the peak value.

### Data collection

All patients who underwent CB ablation of AF were routinely collected clinical research data, including sex, age, past medical history, electrocardiogram, cardiac ultrasound parameters, total ablation time, total operation time, intraoperative fluoroscopy time, intraoperative fluoroscopy dose, intraoperative contrast agent dosage, follow-up data etc.

### Statistical method

**Figure 5:**
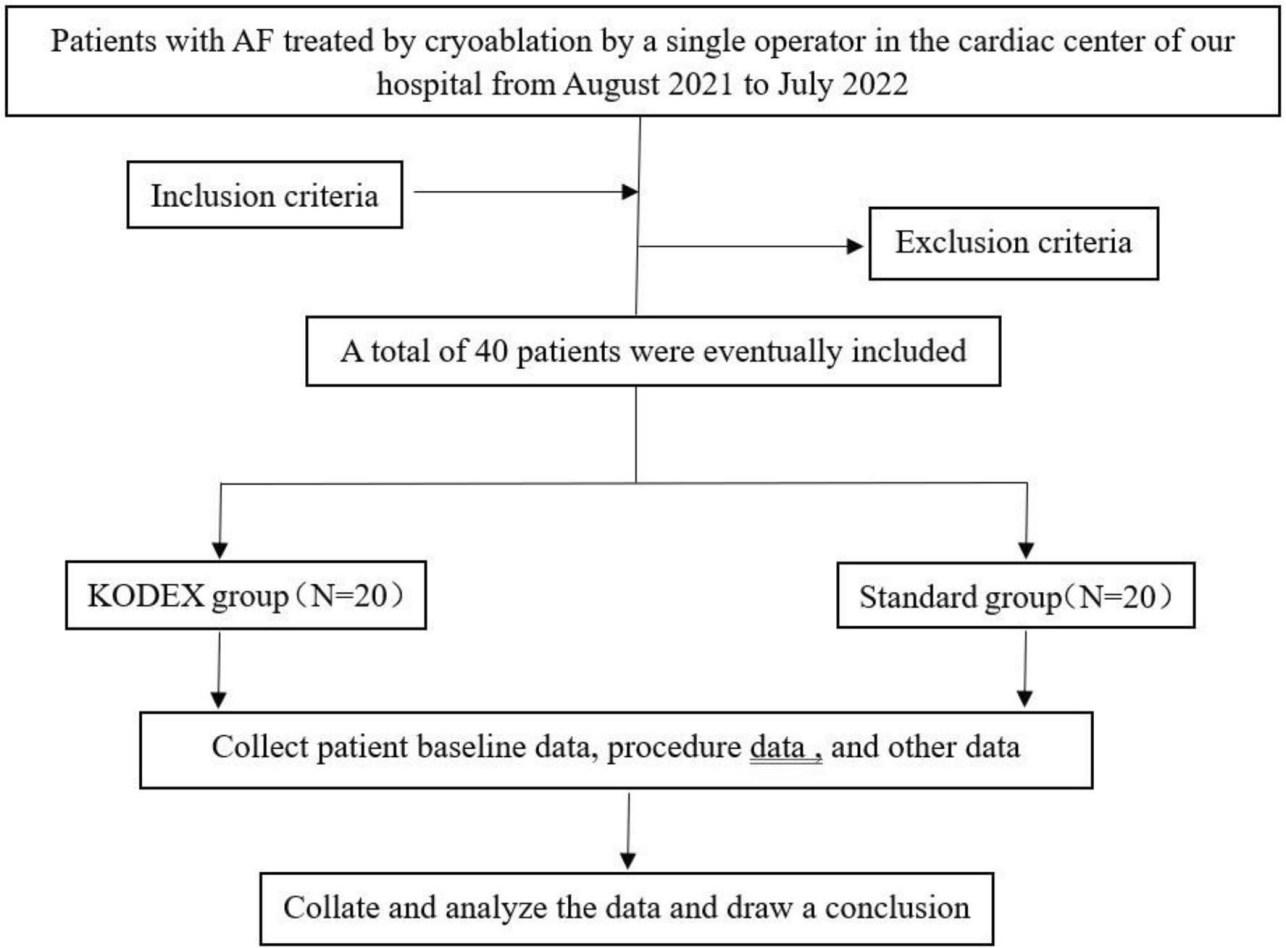
Flowchart of this study.

Continuous variables were described as the as mean ± SD for normally distributed data and median (25–75% quartile) for non-normally distributed data. The comparison between the two groups was performed by two independent samples t-test, and the counting data was analyzed by chi-square test. Using the pulmonary venography as the gold standard, the sensitivity, specificity, positive predictive value (PPV), and negative predictive value (NPV) were calculated including the 95% confidence interval (CI) based on a binomial distribution. All tests were 2-tailed, and a statistical significance was established at P < 0.05. All analyses were performed using SPSS software (version 27.0 SPSS, Inc., Chicago) (Fig. 5).

### Ethical approval

All experimental schemes have been approved by Ethics Committee of the first affiliated Hospital of Xinjiang Medical University and all the procedures being performed were part of the relevant guidelines and regulations (Ethics approval number: 20180622-06).

### Informed consent

All patients understand the indications and risks of the operation before the operation, and sign the operation written consent form. And informed written consent has been obtained from all participants and/or their legal guardians. Besides, this study is carried out in accordance with the principle of the Declaration of Helsinki.

## Result

### Baseline characteristics

A total of 40 patients (27 males and 13 females) were included in this study, mean age was 67.43 ± 10.69 years. The baseline characteristics of the standard group and the KODEX group were shown in Table 1, and there was no significant difference between the two groups.

**Table 1:** Baseline characteristics.

	Conventional group (n = 20)	KODEX group (n = 20)	Statistic quantity	*P*
Gender (male)	15 (75%)	12 (60%)	1.026	0.311
Age (years)	65.10 ± 10.46	69.75 ± 10.66	1.392	0.172
BMI (kg/m^2^)	25.65 ± 3.75	25.25 ± 2.69	0.388	0.700
Hypertension	13 (65%)	12 (60%)	0.107	0.744
Diabetes	3 (15%)	4 (20%)	0.173	1.000
CHD	8 (40%)	6 (30%)	0.440	0.507
Stroke	6 (30%)	2 (10%)	2.500	0.235
Heart failure	1 (5%)	0 (0%)	1.026	1.000
Renal insufficiency	0 (0%)	2 (10%)	2.105	0.487
Creatinine clearance rate (μmol/l)	89.52 ± 22.13	77.86 ± 16.53	1.888	0.067
CHA_2_DS_2_-VASc score	2.20 ± 1.61	2.30 ± 1.46	0.206	0.838
HAS-BLED score	1.15 ± 0.81	1.25 ± 0.64	0.433	0.668
LA (mm)	40.65 ± 4.92	39.85 ± 6.18	0.453	0.653
LV (mm)	50.10 ± 7.77	48.90 ± 7.21	0.506	0.615
LVEF (%)	58.53 ± 8.14	59.50 ± 6.61	0.413	0.682

### Procedure data

20 cases were treated with the KODEX-EPD system and 20 cases with conventional fluoroscopy. The total ablation time and total operation time in KODEX group were (36.40 ± 6.72 min vs 35.15 ± 6.29 min, P > 0.05) and (64.20 ± 11.82 min vs 66.00 ± 13.18 min, P > 0.05), there was no significant difference between the two groups. The intraoperative fluoroscopy time was (532.30 ± 72.83 s vs 676.25 ± 269.33 s P < 0.05), the intraoperative fluoroscopy dose was (110.00 ± 28.64 mGy vs 144.68 ± 66.66 mGy, P < 0.05), and the intraoperative contrast agent dosage was (71.90 ± 5.97 ml vs 76.05 ± 5.93 ml, P < 0.05)in the KODEX group compared with the routine group (Table 2).

**Table 2:** Intraoperative related indicators.

	Conventional group (n = 20)	KODEX group (n = 20)	Statistic quantity	*P*
Vascular puncture (min)	20.90 ± 6.79	20.45 ± 9.65	0.171	0.866
Atrial septal puncture (min)	9.95 ± 5.76	7.35 ± 2.54	1.846	0.076
LSPV ablation (min)	9.75 ± 3.85	10.30 ± 2.68	0.524	0.603
LIPV ablation (min)	6.85 ± 2.08	7.35 ± 2.11	0.754	0.455
RSPV ablation (min	9.20 ± 2.84	9.75 ± 3.81	0.518	0.608
RIPV ablation (min)	8.50 ± 3.65	8.65 ± 2.62	0.149	0.882
SVC ablation (min)	0.75 ± 1.55	0.55 ± 1.36	0.434	0.667
Total ablation time (min)	35.15 ± 6.29	36.40 ± 6.72	0.607	0.547
Total operation time (min)	66.00 ± 13.18	64.20 ± 11.82	0.455	0.652
Fluoroscopy time (s)	676.25 ± 269.33	532.30 ± 72.83	2.307	0.031
Fluoroscopy dose (mGy)	144.68 ± 66.66	110.00 ± 28.64	2.138	0.042
Contrast agent dosage (ml)	76.05 ± 5.93	71.90 ± 5.97	2.206	0.033

### Learning curve comparison

**Figure 6:**
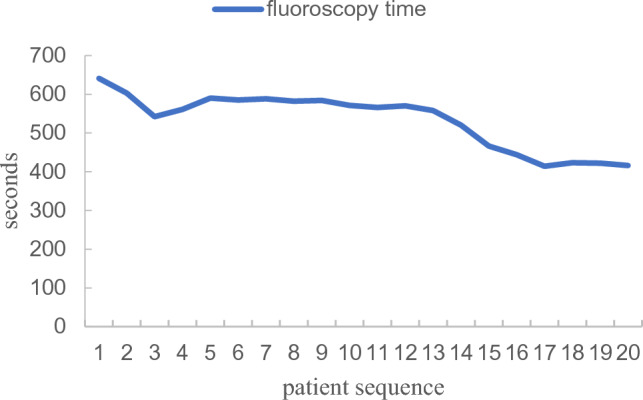
Learning curve of fluoroscopy time.

**Figure 7:**
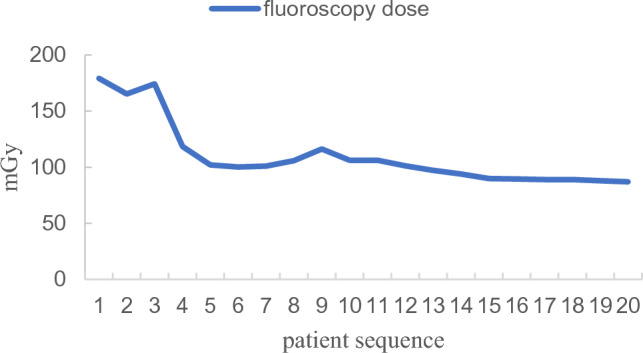
Learning curve of fluoroscopy dose.

**Figure 8:**
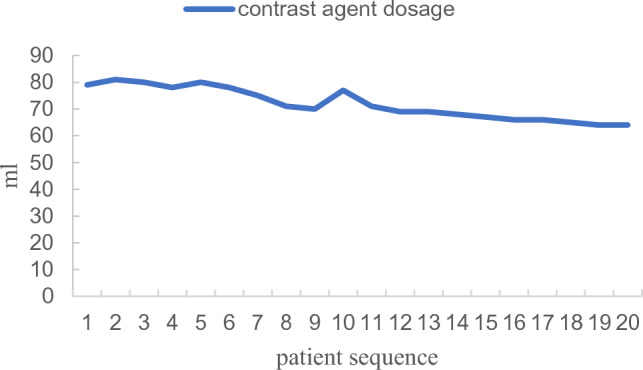
Learning curve of contrast agent dosage.

In the KODEX group, the first 5 patients were compared with the latter 15 patients in terms of the learning curve (Table 3). The total ablation time was (36.27 ± 6.34 min vs 36.80 ± 8.56 min, P > 0.05) and the total operation time was (62.60 ± 13.10 min vs 69.00 ± 5.00 min, P > 0.05). There were significant differences between the two groups in intraoperative fluoroscopy time (513.93 ± 73.02 s vs 587.40 ± 38.34 s, P < 0.05), fluoroscopy dose (97.39 ± 8.80 mGy vs 147.85 ± 35.19 mGy, P < 0.05), and contrast agent dosage (69.33 ± 4.45 ml vs 79.60 ± 1.14 ml, P < 0.05). The trend was shown in Figs. 6, 7, and 8.

**Table 3:** Comparison of learning curve.

	Learning group (n = 5)	Post-learning group (n = 15)	Statistic quantity	*P*
Total ablation time (min)	36.80 ± 8.56	36.27 ± 6.34	0.150	0.883
Total operation time (min)	69.00 ± 5.00	62.60 ± 13.10	1.579	0.133
Fluoroscopy time (s)	587.40 ± 38.34	513.93 ± 73.02	2.883	0.012
Fluoroscopy dose (mGy)	147.85 ± 35.19	97.39 ± 8.80	3.173	0.032
Contrast agent dosage (ml)	79.60 ± 1.14	69.33 ± 4.45	8.166	< 0.001

**Figure 9:**
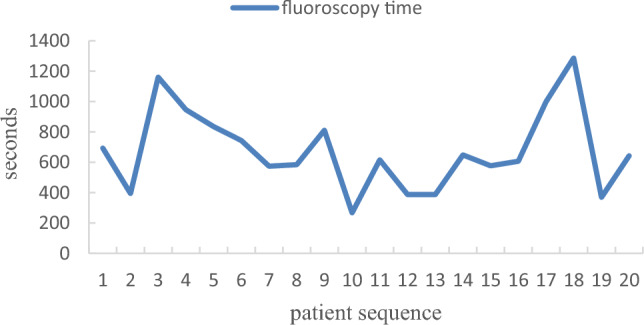
Learning curve of fluoroscopy time.

**Figure 10:**
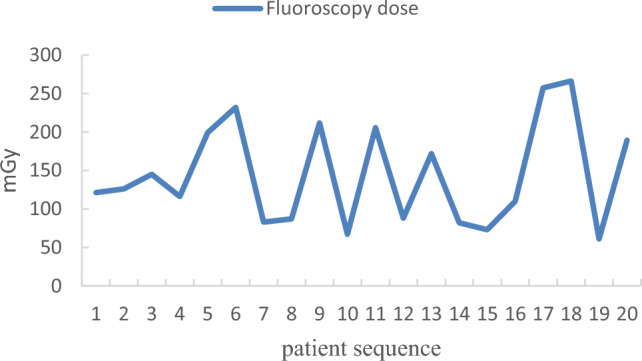
Learning curve of fluoroscopy dose.

**Figure 11:**
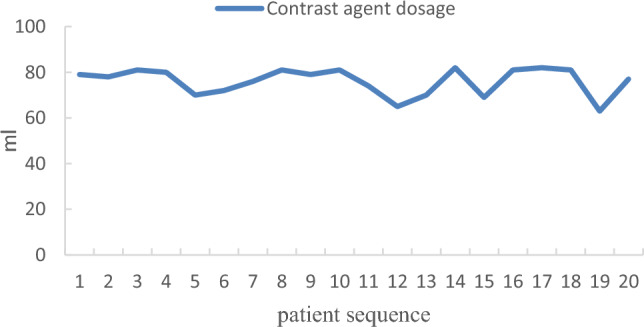
Learning curve of contrast agent dosage.

In the standard group, the first 5 patients were compared with the latter 15 patients in terms of the learning curve (Table 4). There were significant differences between the two groups in intraoperative the total ablation time (41.20 ± 6.91 min vs 33.13 ± 4.76 min, P < 0.05) and the total operation time (80.20 ± 12.70 min vs 61.27 ± 9.68 min, P < 0.05). There were no significant differences between the two groups in intraoperative fluoroscopy time (805.60 ± 286.47 s vs 633.13 ± 258.90 s, P > 0.05), fluoroscopy dose (141.58 ± 33.99 mGy vs 145.72 ± 75.47 mGy, P > 0.05), and contrast agent dosage (77.60 ± 4.39 ml vs 75.53 ± 6.40 ml, P > 0.05). The trend was shown in Figs. 9, 10, and 11.

**Table 4:** Comparison of learning curve.

	Learning group (n = 5)	Post-learning group (n = 15)	Statistic quantity	*P*
Total ablation time (min)	41.20 ± 6.91	33.13 ± 4.76	2.939	0.009
Total operation time (min)	80.20 ± 12.70	61.27 ± 9.68	3.516	0.002
Fluoroscopy time (s)	805.60 ± 286.47	633.13 ± 258.90	1.259	0.224
Fluoroscopy dose (mGy)	141.58 ± 33.99	145.72 ± 75.47	0.167	0.869
Contrast agent dosage (ml)	77.60 ± 4.39	75.53 ± 6.40	0.666	0.514

### Performance of KODEX-EPD injection in evaluating PV occlusion

In this study, 79 PV occlusions in 20 patients with KODEX-EPD were analyzed. All PVs were electrically isolated. Compared to the results of the X-ray evaluation, a total of 78 times (42.8%) were completely occluded, 58 times were small leaks (31.9%), and 46 times were large leaks (25.3%). Overall, when the X-ray results indicate completely occluded, the sensitivity of KODEX-EPD injection tool in predicting the outcome of PV occlusion was 93.6% (95% CI 85–97.6%, P < 0.01), the specificity was 69.6% (95% CI 54–81.8%, P < 0.01), and the positive predictive value was 83.9% (95% CI 74.1–90.6%, P < 0.01). The negative predictive value was 86.5% (95% CI 70.4–90.6%, P < 0.01). The evaluation results of the specific four PVs are shown in Table 5. When the X-ray showed a small leak, the sensitivity of the KODEX-EPD injection tool was 93.1% (95% CI 82.4–97.8%, P < 0.01), the specificity was 82.0% (95% CI 65.9–91.9%, P < 0.01), and the positive predictive rate was 88.5% (95% CI 77.1–94.1%, P < 0.01). The negative predictive value was 88.9% (95% CI 73–96.3%, P < 0.01).

**Table 5:** Sensitivity and specificity of the Injection tool for PV occlusion verification.

LSPV		CL 95%	RSPV		CL 95%
Sensitivity	95.2%	74.1–99.8	Sensitivity	91.7%	59.8–99.6
Specificity	75%	21.9–98.7	Specificity	60%	32.9–82.5
Positive predictive value	95.2%	74.1–99.8	Positive predictive value	64.7%	38.6–84.7
Negative predictive value	75%	21.9–98.7	Negative predictive value	90%	54.1–99.4

LIPV		CL 95%	RIPV		CL 95%
Sensitivity	95.2%	74.1–99.8	Sensitivity	91.7%	71.5–98.5
Specificity	85.7%	42–99.2	Specificity	71.4%	47.7–87.8
Positive predictive value	95.2%	74.1–99.8	Positive predictive value	78.6%	58.5–90.9
Negative predictive value	85.7%	42–99.2	Negative predictive value	88.2%	62.3–97.9

All patients were followed up routinely after operation, with an average follow-up of (9.90 ± 1.06) months. Follow-up standard group and KODEX group for postoperative arrhythmia recurrence and whether to continue taking antiarrhythmic drugs, the follow-up results are shown in Table 6. There was no significant difference in arrhythmia recurrence and antiarrhythmic drugs taking after CB ablation between the two groups (*P* > 0.05). There were no operation-related complications between the two groups.

**Table 6:** Follow-up data.

	Conventional group (n = 20)	KODEX group (n = 20)	Statistic quantity	*P*
Recurrent arrhythmia	6 (30%)	4 (20%)	0.716	0.358
Continue to take antiarrhythmic drugs	9 (45%)	6 (30%)	0.960	0.327

## Discussion

**Figure 12:**
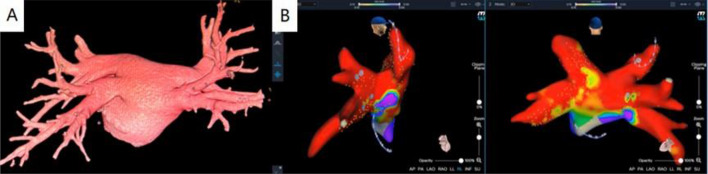
3D reconstruction image of the heart and modeling image of KODEX-EPD mapping and navigation system.

The KODEX-EPD mapping system creates high-resolution cardiac anatomical images by utilizing the unique dielectric properties of biological tissues. When compared to other mapping system, this system has the advantage of non-contact modeling, which can more accurately locate the PV and related accessory structures, increasing the operation's success rate (Fig. 12). Previous studies^15–17^ have pointed out that KODEX-EPD can show complex and abnormal anatomical structures during CB of AF and verify the effect of PVI without injection of contrast agent (baseline tool). KODEX-EPD can generate atrium and PV geometry^18^. Romanov^19^ confirmed the function of KODEX-EPD to accurately detect catheter position and generate high-resolution images in an animal experiment. The results of Maurer et al.^20^ show that the KODEX-EPD mapping system can produce 3D real-time and high-resolution images of left atrial anatomy with comparable CT quality without the use of additional imaging. Furthermore, a study^21^ compared the PV size measured by selective angiography to the PV imaging size measured by KODEX-EPD, and there is no statistically significant difference in the measurement between the two methods. In this study, KODEX-EPD system correctly identified the common trunk of PV, which informed the ablation strategy and guided the precise ablation.

Although the proximal seal technique (PST) is a recommended method to avoid ablation within the tubular portion of the PV, there is no qualitative description of the workflow. The use of PST to achieve the vestibular site of PV ablation without a large leakage has not been well described. Thus, in this study, we assessed the feasibility of using a new mapping system, KODEX-EPD, to standardize PST in a group of patients. During the ablation of PV with PST, the injection curve for the KODEX-EPD system displayed three-stage characteristics as follows: the first part of the curve climbed rapidly; the second part of the curve could maintain a high position or a lower slope; and the third part of the curve decreased slowly and eventually stabilized at about half of the peak value. After our center regularly used it to guide the CB ablation of AF, our data indicated that KODEX-EPD system had good sensitivity and specificity compared to X-ray results when the X-ray results showing complete occlusion. In the case of small leakage of X-ray results, the sensitivity and specificity of KODEX-EPD system evaluation results were 93.1% and 82% respectively, which preliminarily proved the value and potential of the KODEX-EPD 3D navigation system in CB ablation surgery. The sensitivity and specificity of the right pulmonary system is slightly lower than those of the left lung, possibly due to the influence of catheter operation. Because a second-generation Medtronic balloon was used in this study, the catheter operation required more space than a fourth-generation balloon or RF ablation catheter to achieve balloon bending and movement among other operations. In terms of catheter and sheath operation, the right PV requires more catheter and sheath operation, and the Achieve electrode is frequently sent into the depths of the PV to meet the requirements of balloon placement and stable position, which may affect the function of occlusion evaluation based on the dielectric principle.

Fluoroscopy exposure has a significant negative impact on patients and operators, these risks include acute and subacute skin diseases, musculoskeletal injuries, cataracts, malignancies and genetic defects^22,23^, so steps must be taken to reduce exposure to a bare minimum. The continuous development of intracardiac echocardiography and three-dimensional electronic mapping system has changed the workflow of catheter ablation and can be operated under the condition of continuous reduction or even elimination of radiation, further reducing the potential risks associated with radiation exposure of patients and operators^24^. CB ablation guided by KODEX-EPD is proved to be feasible and safe^25^. Prior to our study, our center analyzed patients who underwent permanent pacemaker implantation using the KODEX-EPD 3D mapping system and found that the KODEX-EPD 3D mapping system can significantly reduce intraoperative fluoroscopy time and dose compared to conventional operation^26^. The results of this study show that, when compared to the traditional two-dimensional cryopreservation group, 3D CB ablation under the guidance of KODEX-EPD system does not significantly increase the operation time, but the fluoroscopy time and fluoroscopy dose are significantly reduced, indicating that the use of KODEX-EPD system can reduce the fluoroscopy exposure, which is similar to the results of Schillaci’s investigation^27^.

Some patients with renal insufficiency, elderly patients with heart failure, and contrast agent allergies may be unable to undergo CB ablation. However, according to one study^28^, the KODEX-EPD system can significantly reduce the use of contrast agents in patients. This study also discovered that the KODEX group used significantly less contrast agent than the standard group. This suggests that the KODEX-EPD system has some advantages in terms of reducing contrast agent use. It could avoid deterioration of cardiac and renal function in the aforementioned patients. The indications for CB ablation can be expanded in the future by substituting saline for contrast agent and using the balloon 3D visualization function, while the risks associated with contrast agent injection are reduced.

Our study further investigated the learning curves of the KODEX-EPD system. When compared to the first five patients, the intraoperative fluoroscopy time, fluoroscopy dose, and contrast agent dosage were all significantly reduced. This demonstrates that after completing the learning curve, intraoperative fluoroscopy exposure and contrast agent dosage can be reduced further. Besides, during the medium-and long-term follow-up, there were no operation-related complications. We can conduct longer follow-up to further verify the safety and effectiveness of KODEX-EPD in CB of AF.

As a result, we can conclude that the occlusion evaluation tool has a high sensitivity and specificity for CB ablation. After passing the learning curve, it is possible to reduce fluoroscopy time, fluoroscopy dose, and contrast agent dosage. With the substitution of saline for contrast agent, allowing for 3D visualization of cardiac structure and CB ablation with a low contrast agent dose, the KODEX-EPD system will have high value in the future.

We hope that in the future, KODEX-EPD will be able to provide real-time information about the anatomical structure of the heart and PVs, as well as the location of the balloon, through a simpler and more intuitive graphical interface and more accurate 3D navigation. It can assist operators in correctly evaluating the effect of PVI via green electrophysiology, increasing the success rate of AF CB ablation and decreasing the occurrence of adverse events.

### Study limitations

First, this study was conducted in a single center and included a small number of patients. This might limit the universality of research results. A larger, prospective, multicenter study could shed more light on the role of KODEX-EPD in improving AF treatment via CB ablation. Second, in this study, operators can master the KODEX-EPD system skillfully through the learning curve of 5 cases, and there may be differences among different operators in different centers. Third, this experiment cannot blind the operator, so there may be bias.

## Conclusions

Using the new 3D mapping system KODEX-EPD in CB ablation of AF, compared to the traditional procedure, may significantly reduce radiation exposure and contrast agent use without significantly increasing operation time, making it a feasible method. The occlusion evaluation tool has high sensitivity and specificity in CB ablation and can help with AF CB ablation.

## Data Availability

The datasets generated during and/or analyzed during the current study are available by request.
